# Could molecular variations be predictive in right and left colon cancer in different gender and age groups?

**DOI:** 10.1371/journal.pone.0351228

**Published:** 2026-07-10

**Authors:** Hatice Cilem Solak, Nazli Mert, Asim Leblebici, Zerrin Isik, Ender Berat Ellidokuz, Yasemin Basbinar

**Affiliations:** 1 Department of Translational Oncology, Institute of Oncology, Dokuz Eylul University, Izmir, Turkiye; 2 Department of Gastroenterology, Health Sciences University - Tepecik Education and Research Hospital, Izmir, Turkiye; 3 Department of Information Technologies, Izmir Institute of Technology, Izmir, Turkiye; 4 Department of Computer Engineering, Faculty of Engineering, Dokuz Eylul University, Izmir, Turkiye; 5 Department of Gastroenterology, Faculty of Medicine, Dokuz Eylul University, Izmir, Turkiye; Sichuan University, CHINA

## Abstract

**Background:**

Left-sided colon-cancer (LCC) and right-sided colon cancer (RCC) harbor different clinical entities and different therapy protocols. Our study aimed to demonstrate genomic expression differences and clarify the clinical differences between LCC and RCC by using *in-silico* methods.

**Methods:**

Our study compares mRNA expression levels between clinical groups to describe variations in molecular characteristics between right-left-sided colon cancer (216 right-141 left) using the TCGA-COAD database. We identified differentially expressed genes and analyzed survival profiles for RCC and LCC. We applied a gene set enrichment analysis and merged the results by group to highlight common and different pathways.

**Results:**

The results reveal that TG, INSL5, EREG and AIRE were found as age-related genes, and TG, INSL5, EREG, AIRE, HOXB8 and FLT3 were found as gender-related genes for RCC and LCC. We found that high expression of the AIRE gene was linked with poor survival in patients with RCC in males and under 65 age groups. High expression of LEP was correlated with poor survival in patients with RCC, regardless of gender. In addition, low expression of the EREG gene was linked with poor survival in women with RCC.

**Conclusions:**

Three genes (AIRE, LEP, EREG), which are effective on the EGFR pathway, would have great predictive and prognostic importance in the context of anti-EGFR therapies.

## 1. Introduction

Colorectal cancer (CRC) is one of the most common cancers regardless of gender according to the WHO. WHO-GLOBOCAN 2020 data reveals that CRC ranks third in CRC incidence and second in mortality. Furthermore, CRC is the 2nd and 3rd most common cancer in women and men, respectively [1]. Commonly, the cecum, hepatic flexure, ascending, and transverse colon are defined as the right colon; splenic flexure, descending, and sigmoid colon are defined as the left colon. Right and left colon tumors have different embryological origins (right colon midgut, left colon hindgut), different microenvironments, and blood circulation.

CRC conventional treatment regimens include 5-Fluorouracil (5-FU) and combination chemotherapy with the platinum-based agent, oxaliplatin (FOLFOX), or the topoisomerase inhibitor, irinotecan (FOLFIRI) [2]. These adjuvant chemotherapy regimens; it is more effective in patients with CNS/dMSI and, therefore in patients with LCC [3,4]. In the case of metastases, “targeted” therapies are used alongside conventional regimens to improve survival rates (REF). Aflibercept, bevacizumab, ramucirumab, and regorafenib particularly target the angiogenic pathways, while cetuximab and panitumumab target the EGFR (Epidermal growth factor receptor) pathway [2]. Better responses are obtained in LCC in these targeted therapies applied with conventional therapies [5]. MSI-high tumors are predominantly located on the right side.

Right and left colorectal cancers show different molecular features. DNA mismatch repair mutations and silencing (such as MLH class genes) in RCC and chromosomal instability (CIN) – linked mutations (as APC, KRAS, PIK3CA, p53) are seen in LCRC. Ultimately, these differences shape the course, survival, and treatment of the disease. While LCC patients respond well to adjuvant chemotherapies such as 5-FU-based targeted therapies (anti-EGFR), and progress with a better prognosis, RCC patients do not respond well to standard chemotherapy, but encouraging results are obtained with immunotherapies [6].

Studies have shown that RCCs present at an older age, in females, and with larger tumor sizes, advanced tumor stages, and worse differentiation. In the meta-analysis of nearly 1.5 million patients, including sixty-six studies; at the end of the 65-month follow-up period, left colonic CRCs were related to a reduced risk of death (HR, 0.82; 95%CI (0.79–0.84)) [7]. Nonetheless the underlying molecular events related with the significant difference between LCC and RCC are not well understood. A study was extensively characterized by combining epigenetic (DNA methylation) profiles with expression, methylation, and clinical data of TCGA.

It is thought that there may be vigorous differences at the molecular level in the right and left colorectal cancers. The aim of this study is to discover the molecular differences of RCC and LCC based on gender and age parameters by using in-silico analysis methods and to identify the spatial markers for CRC.Our results would lead researchers to more effectively classify the patients and design new treatments to provide more targeted CRC treatment.

## 2. Materials – methods

The analysis was carried out in the R-Bioconductor environment. Ethical approval and consent to participate were not applicable to this study.

### 2.1. Data selection

TCGA-COAD (The Cancer Genome Atlas - COlorectal ADenocarcinoma) RNA-sequencing data were used as the main patient cohort. ‘Primary solid tissue’ samples were selected out of available patient samples. Multiple samples with the same patient identifier were eliminated. Those with ascending, cecum, hepatic flexure, and transverse colon were assigned as the right side; those with descending, splenic flexure, sigmoid colon, and rectosigmoid junction were assigned as the left-sided.

### 2.2. Data analysis

The data were downloaded by using the TCGAbiolinks package [8]. The downloaded data was prepared for analysis by applying the TCGAanalyze_Preprocessing function. A square symmetric spearman correlation matrix is calculated to find low correlation samples with outliers between samples (cor.cut = 0.6) and TCGAanalyze_normalization function. Reads were normalized within and between lanes using default parameters, following the EDASeq R package [9] instructions. Genes having statistically significant expression changes were calculated. The adjusted p-value was selected as < 0.05 and the fold-change of |logFC|>=1 was applied. Significant changes were calculated when comparing gene expressions of left-sided and right-sided colon samples for the selected clinical subtypes. The differentially expressed genes (DEG) were created as the result of this analysis.

### 2.3. Gene set enrichment analysis

Enrichment analysis was applied to the DEG obtained as a result of the data analysis. EnrichR [10] and Cytoscape 3.9.1 [11] were used for this analysis and visualization purposes.

### 2.4. Survival analysis

The maximally selected rank statistics method in the survminer package was used to separate the group according to gene levels. Survival [11] and survminer [12] R packages were used for survival analysis.

### 2.5. Protein-Protein Interaction (PPI) analysis

Age- and gender-dependent DEG were determined by PPI analyses. The String.db was used for protein-protein interaction data and the high confidence (≥0.7) interaction score was used to filter the interactions between proteins. Cytoscape 3.9.1 was used to prepare the network figures.

### 2.6. Single nucleotide polymorphism (SNP) and Copy Number Variation (CNV) analysis

Single nucleotide polymorphism (SNP) mutations in signature genes have been investigated to understand age- and gender-dependent mutation in tumors. Pathways of relevant mutations were investigated by CNV of signature genes. GSCALite, a web-based platform, was used for genomic cancer analysis of age- and gender-dependent DEG [13,14].

### 2.7. Drug sensitivity analysis

The relationship between drug sensitivity and DEG, which are crucial for both the right and left colon, in terms of age and gender, was examined using GSCA Lite [13]. The drug sensitivity data obtained from the GDSC and CTRP databases with colorectal cancer drugs used in clinical treatments were subjected to evaluation. The age- or sex-related gene expression data were subjected to a Spearman correlation test.

## 3. Results

### 3.1. Patient & tumor characteristics

**Fig 1:**
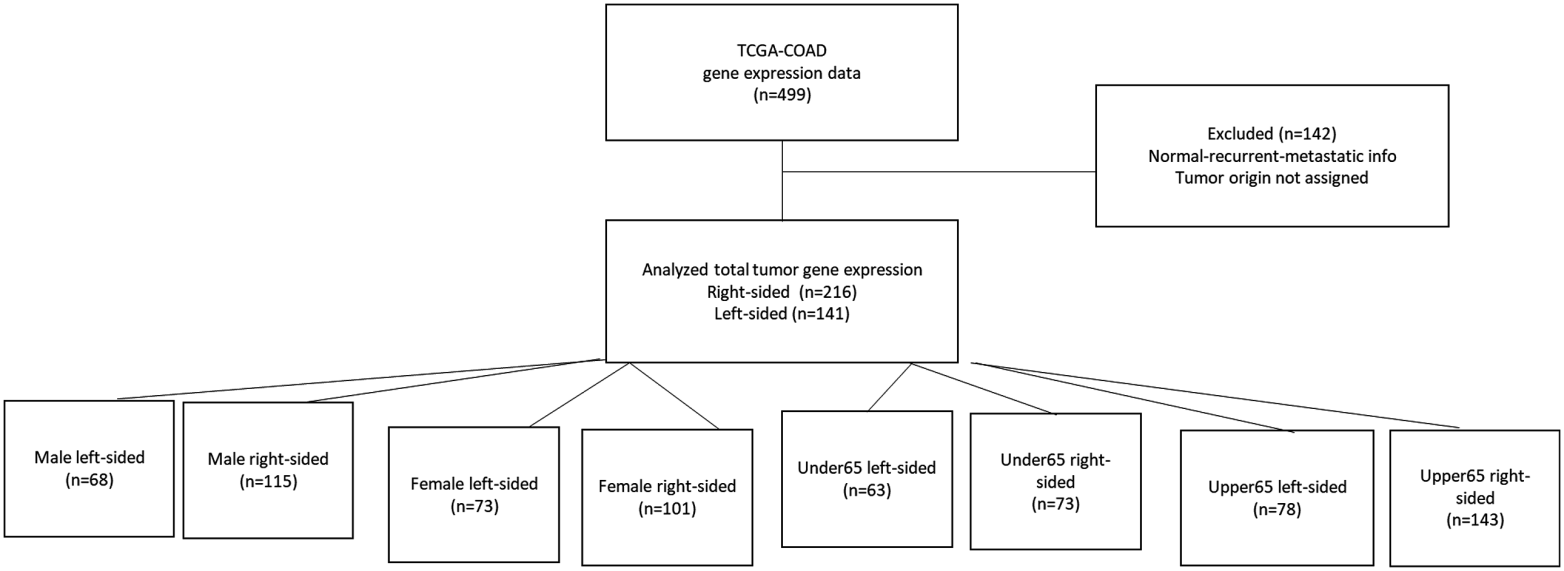
The workflow and information of the groups included in the study.

Gene expression data including 41 normal, 478 primary, 1 recurrent, and 1 metastasis were extracted from the TCGA-COAD project. Normal, recurrent, metastatic, multiple samples from the same patient and those without tumor extraction site information were excluded. The workflow and information of the groups included in the study are given in Fig 1.

RCC gene expression data of cecum (95), ascending (84), hepatic flexura (15) and transverse (22) regions were distributed according to groups as 115 males, 101 females, 73 samples under 65 years old and 143 samples over 65 years old. LCC gene expression data, defined from splenic flexure (5), descending (17), sigmoid (112), and rectosigmoid (7) regions are distributed according to the groups as 68 males, 73 females; 63 samples under age 65 and 78 samples over age 65 (S1 and S2 Table).

### 3.2. Gender specific pathway enrichment results

Enriched Kegg pathways of genes, whose expression changes significantly different in the left-sided compared to the right-sided, were obtained based on the gender groups (S1 Fig).

Neuroactive ligand-receptor interaction terms are common to both male and female groups:

- In males, the expressions of the NPW, LPAR3, PYY, P2RY4, PRSS2, INSL5, and NTS genes were higher in the left colon compared to the right. Conversely, the expressions of CHRNA4, LEP, DRD5, CCK, PTH2R, GABRP, GCGR, CTSG, PTGDR, GRIK2, CNR2, and CNR1 genes in the right colon were higher than in the left side.
- In females, the expressions of the GRM8, NPFFR1, GABRQ, DRD1, KNG1, GABRR1, PYY, SCT, TAC1, GRPR, DRD2, INSL5, LEP, and GPR83 genes were higher in the left colon compared to the right. On the other hand, the expressions of CALCB, DRD5, CHRND, PRSS2, CCK, PTH2RRNA, NMUR2, CH7, GABRP, CHRM1, ADRB1, GZMA, FPR2, OPRD1, CHRM4, MTNR1A, CTSG, KISS1R, HRH2, PTGER2, GIPR, and AVPR1AABC genes in the right colon were higher than in the left side.

The pathways identified only in the female group were ABC Transporters, African trypanosomiasis, Arachidonic acid metabolism, Bile secretion, Cytokine-cytokine receptor interaction, Fat digestion and absorption, Graft-versus-host disease, Linoleic acid metabolism, Mineral absorption, Natural killer cell-mediated cytotoxicity, Pancreatic secretion, Protein digestion, and absorption, Serotonergic synapse. The pathways identified only in the male group were Autoimmune thyroid disease, Hematopoietic cell lineage, Primary immunodeficiency.

**Fig 2:**
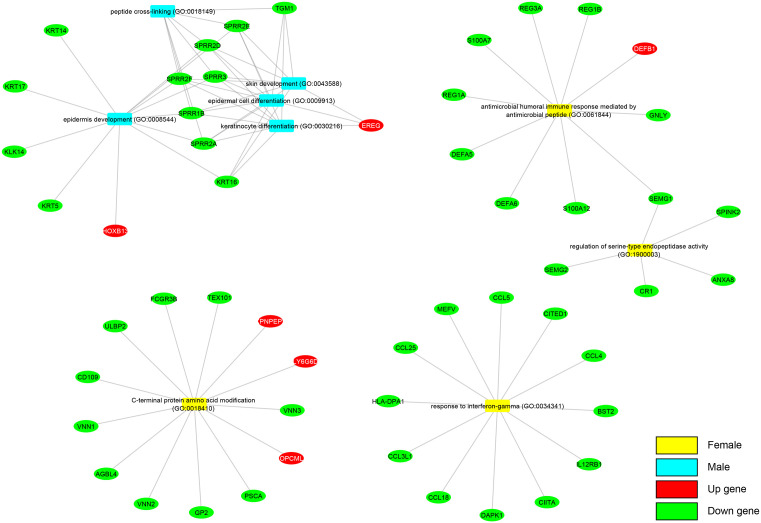
The pathways identified in both genders.

The Gene Ontology (GO)-Biological Process (BP) terms of the female group covered antimicrobial humoral immune response mediated by antimicrobial peptide (GO:0061844), C-terminal protein amino acid modification (GO:0018410), Regulation of serine-type endopeptidase activity (GO:1900003), Response to interferon-gamma (GO:0034341). Furthermore, GO-BP terms for male group resulted epidermal cell differentiation (GO:0009913), epidermis development (GO:0008544), keratinocyte differentiation (GO:0030216), peptide cross-linking (GO:0018149), skin development (GO:0043588). The results are given in Fig 2.

The Cancer Hallmark terms of genes were obtained based on the gender groups (S2 Fig). The term “KRAS Signaling Down” has become common to both male and female groups.

- In the male group, the expressions of the TG, P2RY4, CLDN8, and INSL5 genes were higher in the left colon compared to the right. Conversely, in the right colon, the expressions of TFAP2B, SPRR3, KRT5, COL2A1, PAX4, KRT13, CDH16, TCL1A, GP2, KCNQ2, KCNN1, and TGM1 genes were higher than in the left side.
- In the female group, the expressions of the TENM2, CKM, FGGY, CLDN8, SLC38A3, and INSL5 genes were higher in the left colon compared to the right. On the other hand, in the right colon, the expressions of CALCB, TFF2, GP2, KRT5, COL2A1, PNMT, SERPINB2, SPRR3, KLK7, SLC16A7, KRT1, WNT16, ABCB11IF, ZNGBTB16, PRODH, PDCD1, MEFV genes were higher than in the left side.

The terms “Allograft Rejection” and “Pancreas Beta Cells” were found only in the female group. The term “KRAS Signaling Up” was found to be involved only in the male group.

### 3.3. Age specific pathway enrichment results

Neuroactive ligand-receptor interaction Kegg pathway is also common in both under / over 65 age groups was given in S3 Fig.

- In the over-age of 65 group, the expressions of the GRM8, ADRA1D, DRD2, DRD1, GRPR, SCT, PYY, INSL5, NTS, and GCG genes were higher in the left colon compared to the right. Conversely, in the right colon, the expressions of DRD5, CALCB, CCK, PTH2R, GABRP, CHRNA7, GCGR, PTGDR, KISS1R, GZMA, MTNR1A, and CHRM1 genes were higher than in the left side.
- In the under age 65 group, the expressions of the TAC1, DRD2, INSL5, and GPR83 genes were higher in the left colon compared to the right. On the other hand, in the right colon, the expressions of CHRNA4, DRD5, CHRND, GCG, OPRD1, CHRNB2, SST, CTSG, NMUR2, GABRP, GRIK2, CHRM1, CHRNA7, NTSR1, CNR2, SSTR3, GZMA, GRIN1, HRH2, and P2RY8 genes were higher than in the left side.

Primary immunodeficiency terms were only found to be involved in the group under-65 age. Linoleic acid metabolism, Pancreatic secretion, Salivary secretion, and Steroid hormone biosynthesis terms were only found in the over-65 age groups.

The “KRAS Signaling Dn” term in the cancer hallmarks has become common in both under / over 65 age groups was given in S4 Fig.

- In the over-age of 65 group, the expressions of the CKM, TG, SLC38A3, INSL5, and CLDN8 genes were higher in the left colon compared to the right. Conversely, in the right colon, the expressions of CALML5, GP2, SPRR3, COL2A1, CALCB, KRT13, TFF2, PDCD1, PRODH, and IFNG genes were higher than in the left side.
- In the under age 65 group, the expressions of the FGGY, INSL5, and SERPINA10 genes were higher in the left colon compared to the right. On the other hand, in the right colon, the expressions of TFAP2B, KRT5, SPRR3, TFF2, PAX4, PNMT, TCL1A, KCNQ2, PAX3, SERPINB2, COL2A1, KRT1, KCNN1, ABCB11, TGM1, RYR1, LYPD3, SLC6A14, SLC16A7, and PDCD1 genes were higher than in the left side.

The terms Allograft Rejection, Complement, KRAS Signaling Up, and Pancreas Beta Cells were found to be involved only in the under 65 age group.

**Fig 3:**
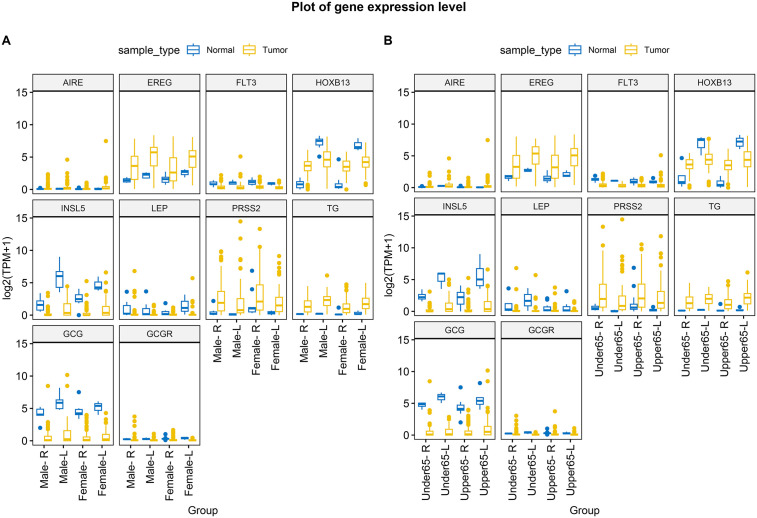
The gene expression differences in normal and tumor tissues according to gender and age groups.

The gene expression differences in normal and tumor tissues according to gender and age groups were given in Fig 3. Boxplots were grouped based on the sample location by taking the logarithm of the Transcript per million (TPM) expression value. Fig 3 shows the conditions with location according to the normal and tumor tissue. The expression levels of INSL5 and GCG genes in tumor tissue were lower than normal tissue. Furthermore, the HOXB13 gene was expressed at higher levels in the right-sided tumor compared to normal in all groups, while the opposite was observed for the left-sided tumor.

### 3.4. Survival analysis of significant genes

As a follow up study, we applied a survival analysis of important genes identified by pathway enrichments. High expressions of AIRE, LEP, and TG genes, respectively, were associated with worse overall survival (OS) in colon tissue of male patients (S5 Fig-(a1-2–3)). Hazard ratios for AIRE, LEP, and TG were found as HR 1.9 [95% CI: 1.01–3.7], p = 0.048; HR 2.5 [95% CI: 1.34–4.5], p = 0.005; HR 4.6 [95% CI: 0.98–21.2], p = 0.054, respectively (S5 Fig-(a4). When the relationship between the expression levels of AIRE, LEP, and TG genes and survival rates was analyzed by colon region, a poor survival association was found with high expression for both right and left colon.

In females, low expression level for the EREG gene was associated with worse survival (S6 Fig) On the other hand, those female patients with high expression levels for the LEP gene were associated with poor survival in S6 Fig-(a2), respectively, Hazard ratios for EREG and LEP were found as HR 2.6 [95% CI:1.2–5.4], p = 0.013; HR 2.9 [95% CI:1.4–5.9]; p = 0.004, respectively and given in S6 Fig-(a3). When the relationship between the expression levels of EREG and LEP genes and survival rates was analyzed by the colon region for female patients, a poor survival association was found with low expression of EREG for the right side and a poor survival association was observed with high expression of LEP for the right side.

High expression of AIRE was found to be correlated with worse overall survival (OS) in patients under 65 years old as in S7 Fig-(a1), (HR 2.7 [95% CI:1.2–5.8], p = 0.013; S7 Fig-(a2)). When the relationship between the expression level of the AIRE gene and survival rate was analyzed by the colon region for patients under 65 age, a poor survival association was observed with high expression of AIRE for the right side.

**Fig 4:**
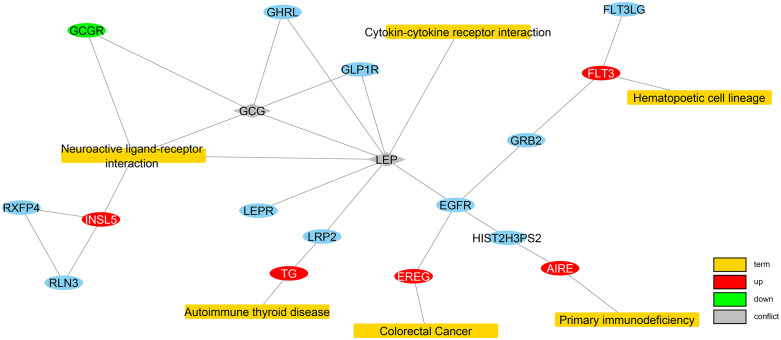
The pathway enrichment of DEG, the PPI network, and the Kegg pathways.

Low expression of GCG was found to be correlated with worse OS in patients over 65 years olds as in S8 Fig-(a1), (HR 2 [95% CI:1.2–3.4], p = 0.011; S8 Fig-(a2)). When the relationship between the expression level of the GCG gene and survival rate was analyzed by colon region for patients over 65 years old, a poor survival relationship was found with low expression of GCG for the right side. The pathway enrichment of DEG, the PPI network, and the Kegg pathways were given in Fig 4. AIRE, EREG, FLT3, GCG, GCGR, HOXB13, LEP, TG genes were found to be effective in the EGFR-related pathway.

### 3.5. GSCA

**Fig 5:**
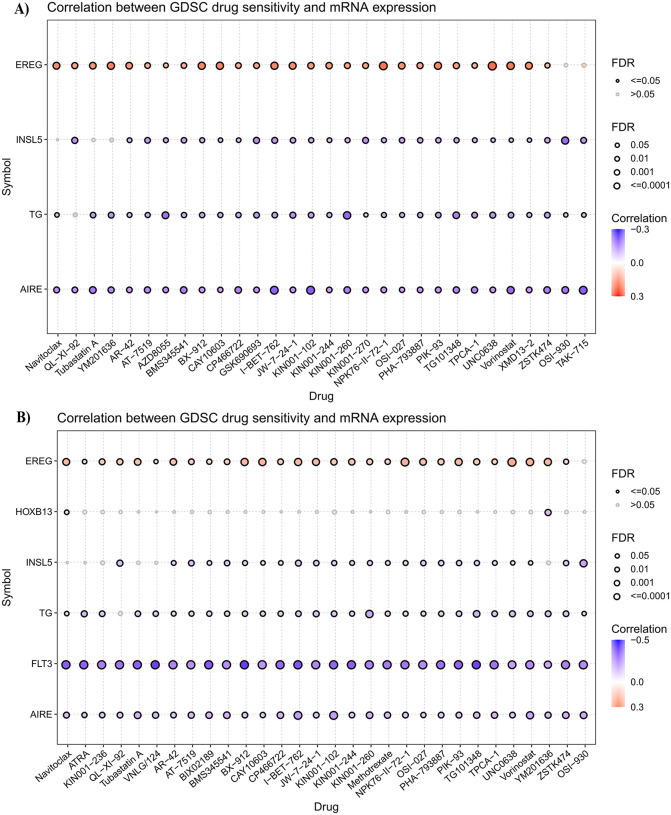
Drug sensitivity analysis based on the expression of a) Age-related b) Gender related genes using the GDSC database.

The study was conducted to investigate the correlation between drug sensitivity and mRNA expression of age- and gender-related genes of sidedness of CRC, with a particular focus on EGFR-therapies. According to the results of GDSC analysis, EREG gene was positively correlated to all drugs, while LEP, FLT3 and AIRE genes were negatively correlated to all drugs. The results suggest a correlation between gene expression and drug sensitivity, with potential implications for understanding and overcoming drug resistance in EGFRs (Fig 5a-b).

**Fig 6:**
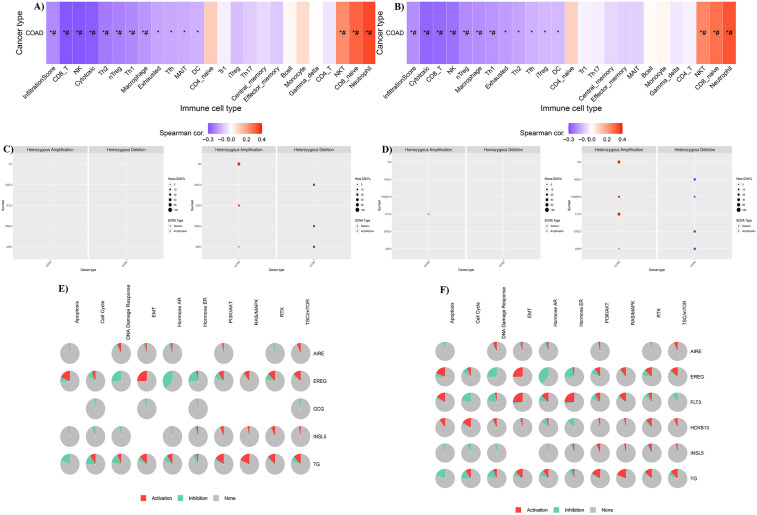
GSCALite platform for tumor infiltration, CNVs and pathway enrichment analysis of age and gender-related genes. Tumor infiltration results for A) age-related and B) gender related genes. Homozygous and heterozygous CNVs for C) age-related and D) gender-related genes. Venn diagram of E) age-related and F) gender-related genes using functional enrichment pathways.

The infiltration results of age and gender-related genes of CRC revealed that neutrophil and nTreg cells were found to have the highest and the lowest infiltered cells, respectively. Homozygous copy number variations (CNVs) are particularly significant genetic alterations that can affect tumour behaviour and response to treatment. The identification of these CNVs can provide insights into the pathways contributing to tumour formation and the tumour’s responsiveness to therapies, thus enabling the selection of targeted treatments and improving patient outcomes [15]. Pathway enrichment analysis based on GeneSet Variation Analysis (GSVA) algorithm showed that age and gender related genes active in various immune-related pathways of COAD. All results were given in Fig 6.

## 4. Discussion

This study analyzed the molecular characteristics (similarity and difference in mRNA expression data and survival profiles) of a total of 357 colon cancer samples and identified spatial gene markers for right- and left-sided CRC based on gender and age parameters. AIRE, LEP, and TG were important markers for side-dependent, gender, and age. These genes would have clinical relevance for targeted therapy when results of pathway analyses are considered.

Regarding right versus left colon cancer, there are several key differences in gene expression and clinical outcomes. Right-sided colon cancer is linked with a higher frequency of BRAF mutations and microsatellite instability, while left-sided colon cancer is more commonly associated with KRAS mutations [6]. From a clinical perspective, different outcomes are seen in the use of anti-EGFR agents. Up-to-date guidelines recommend the use of cetuximab or panitumumab in wild-type RAS and particularly left-sided colon cancers. The expected benefit is not seen in right colon cancers. Predictor factors that will predict anti-EGFR treatments, including left-sided colon cancer and KRAS wild type, have not been clarified yet [16].

AIRE, EREG, FLT3, INSL5, HOXB13, LEP, PRSS2, TG, GCG, and GCGR genes, which we found important because of the significant expression analysis, integrative pathway analysis on gender and age groups, survival analyses, and protein-protein interaction analysis.

AIRE (Autoimmune regulator) is a self-tolerance transcriptional factor, functioning in the thymus. It regulates the presentation of a wide range of self-antigens in T cell maturation and clonal deletion processes. It is also expressed in tolerogenic antigen-presenting cells in secondary lymphoid organs such as gut-associated lymphoid tissue and Peyer’s patches. Moreover, extrathymic expression has been observed in several cancer types including breast, prostate, and oral squamous cell carcinoma [17,18]. It is often related to poor prognosis in malignancies and auto-immune diseases. This study has shown that AIRE expression is significantly higher in right-sided colon cancer than in left-sided colon cancer for all comparison groups. For the male and under-65 groups, those with high AIRE gene expression had poor survival. Additionally, we found that those with high AIRE gene expression on the right had worse survival than those with high on the left side.

Our finding that AIRE overexpression is associated with poorer survival in under-65 male RCC patients is consistent with AIRE’s capacity to reinforce peripheral immune tolerance and thereby facilitate tumour immune evasion. However, emerging evidence indicates that reduced AIRE function may contribute to carcinogenesis through a different pathway. In the thymus, AIRE is essential for central tolerance; when its expression or activity is diminished, autoreactive T cells escape, leading to chronic inflammation that preferentially affects the right colon. Recent studies have shown that autoimmune conditions, particularly inflammatory bowel disease, confer a 1.5–3.1-fold increased risk of CRC, with a striking predilection for right-sided tumours, and that this association is most pronounced in younger males and older females [19,20]. Similarly, the literature on repurposing of autoimmune therapies (methotrexate, JAK inhibitors, anti-TNF agents) in CRC suggests potential differential efficacy by age, gender, and sidedness, although prospective data remain limited. These observations provide a coherent biological framework for our results and highlight the need for future studies that integrate AIRE status with autoimmune signatures and therapeutic response in RCC cohorts.

The evidence is remarkably consistent: autoimmune conditions, particularly inflammatory bowel disease (IBD), are associated with a 1.53–2.87-fold increased risk of colorectal cancer, with a significantly higher risk in RCC than in LCC. This association is strongest in patients under 65 years and shows distinct gender patterns — more pronounced in younger males and older females. These relationships remain significant even after adjustment for tumour stage and MSI status [19–21].

The therapeutic armamentarium of inflammatory bowel disease (IBD) provides valuable insights into how immunomodulation may influence colorectal cancer (CRC) risk, particularly with respect to tumour sidedness. JAK inhibitors (e.g., tofacitinib) and anti-TNF agents (infliximab, adalimumab) have well-established safety profiles in large IBD cohorts and do not appear to increase CRC risk; some studies even suggest a potential chemopreventive effect in chronically inflamed right‑sided colonic mucosa. Methotrexate, a conventional immunomodulator, has shown signals of greater efficacy in younger male patients and in right‑sided disease in retrospective analyses, hinting at site‑specific pharmacological activity. Although long‑term data for newer agents such as ustekinumab and vedolizumab are still emerging, their gut‑selective mechanisms—IL‑12/23 blockade and lymphocyte trafficking inhibition—align closely with the distinct immune microenvironment of right‑sided colon cancer (RCC). Collectively, these observations support the hypothesis that IBD therapy may not only mitigate cancer risk but also shape its topographic distribution, reinforcing the need for integrated, side‑specific risk stratification in future research and clinical practice.

EREG (Epiregulin) is a growth factor that activates the EGFR and has been shown to be upregulated in colon cancer. It is known to promote cell growth and proliferation and has been implicated in several types of cancer [22]. Remarkably, a higher expression level of EREG in colon cancer with wild-type KRAS is associated with better effectiveness of therapeutic treatment. On the contrary, the resistance to anti-EGFR treatment in colon cancer was driven by low EREG expression, abnormal genetic mutation, and changes in the signaling pathway. TME plays an important role in the development of different kinds of cancer and resistance to drugs. The control of EREG/EGFR pathways is based on clear mutations of the oncogenic driver and contexts of cells that allow particular pharmacological targeting alone or combinational treatment for tailor-made therapy. New strategies targeting EREG-EGFR, tumor-linked macrophage, and other oncoprotein activation are under development or clinical trials underway [23].

A study showed that OS was seriously cut down in colon cancer patients with down-regulated EREG (HR: 1.7, p = 0.021), which was disclosed as an independent prognostic factor for decreased OS. Our results show that EREG increased on the left side compared to the right side in all groups. In terms of survival data, for the female group, those with low EREG gene expression had worse survival. These patients were evaluated depending on the right and left side to compare how low EREG gene expression is affecting OS on the right and left side tumors. Results show that low EREG gene expression could play a crucial role in right-sided tumors which cause worse OS [24].

LEP is a hormone that regulates energy balance and has been linked to the development of obesity-linked colon cancer. LEP (Leptin) is a gene that encodes a hormone involved in regulating body weight and energy balance. Leptin has also been linked to the development and progression of several types of cancer, including colon cancer. The LEP gene was found to be over-expressed in colon cancer tissue and linked with poor prognosis in patients with colon cancer. It was found that silencing the gene significantly increased the sensitivity of colon cancer cells to the chemotherapeutic agent 5-FU [25]. In another colon cancer study, 9 genes together with the LEP gene were defined as a high-risk group with worse survival outcomes associated with the tumor microenvironment [26]. In our results for the LEP gene, we found that while it was higher on the right for the male group, it was the opposite for the female group. Again, in a study using TCGA and GEO databases, it was reported that the group with high expression of the LEP gene had worse survival than the group with low expression [27]. In our results, we found that high expression of the LEP gene in the male and female groups was associated with poor survival, and when we evaluated this in terms of colon location, it was found to be higher on the right side riskier than on the left.

LEP gene was expressed more on the left side of the female, while it was expressed more on the right side in the male. We showed that while the GCG gene was more on the right side of the under-65 groups, it was more expressed on the left side of the over-65 group.

This study looked at up-regulated genes in the TCGA and GSE-14333 data sets. Among these, the most important down-regulated genes were HOXC6, while Prostate Cancer Susceptibility Candidate (PRAC) and HOXB13 were the most important up-regulated genes in the RCRC compared to the left. It was found to be associated with hypermethylation of DMR in RCRC [3].

Left sided and right sided colorectal tumors have an antigenic nature and an exceedingly active lymphocytic microenvironment. Even if there is an immunogenic load, these tumor cells were not affected by our immune defense. Immune checkpoint regulators, programmed death protein1 (PD-1), ligand version of PD1 (PDL1), and T lymphocyte-related antigen four suppress T-cell activation. Normally, autoimmunity requires immune-checkpoint regulators, but these regulators were used by tumor cells at a high rate as an antitumor immune reaction [40]. In general, immunotherapies are the treatment option in CRCs with hMSI resistant to conventional chemotherapies, unresectable or metastatic CRC [28].

As a summary, AIRE, EREG, and LEP genes were identified by combining expression changes, survival analyses, gene set enrichment, and protein-protein interaction analysis.

The genes discussed are effective in the EGFR pathway and can be evaluated as predictive and prognostic factors within the scope of anti-EGFR treatments after more patient-based prospective studies are performed.

It is under consideration that there might be vigorous molecular differences in right- and left-sided colon cancers and those differences may contribute to success in personalized medicine by revealing new targeted treatment options. Thus, research on this perspective needs to be deepened.

## 5. Conclusion

The objective of our study is to investigate the application of personalised treatments by determining the molecular and immunological changes in genes with age- and sex-dependent differences in expression levels in right-sided colorectal cancer. The findings of our study, which employed informatics tools with a translational approach, have identified potential biomarkers with clinical significance. We concluded that these genes, which are effective in the EGFR-related pathway, should be evaluated as predictive and prognostic factors within the scope of anti-EGFR treatments.

### Main Points:

- Gender and age groups are of great importance in genetic differences for LCC and RCC.
- AIRE, LEP, and EREG show significant differences in gender and age groups.
- EGFR pathway-based genes (AIRE, LEP, and EREG) can be evaluated as important biomarkers of both LCC and RCC.

## Supporting information

S1 Fig Combined results for the KEGG pathway of genes with differential expression in the right-left colon for male and female groups. (PDF)

S2 Fig Combined results for the Cancer Hallmark pathway of genes with differential expression in the right-left colon for male and female groups. (PDF)

S3 Fig Neuroactive ligand-receptor interaction Kegg pathway is also common in both under / over 65 age groups. (PDF)

S4 Fig The “KRAS Signaling Dn” term in the cancer hallmarks has become common in both under / over 65 age groups. (PDF)

S5 Fig Kaplan-Meier survival & cox-regression results in male group colon tissue (a1, a2, a3, a4). Kaplan-Meier survival & cox-regression results (b1, b2, b3, b4) for the right and left side discrimination of the group with poor survival information.
(PDF)

S6 Fig Kaplan-Meier survival & cox-regression results in female group colon tissue (a1, a2, a3, a4). Kaplan-Meier survival & cox-regression results (b1, b2, b3, b4) for the right and left side discrimination of the group with poor survival information.
(PDF)

S7 Fig Kaplan-Meier survival & cox-regression results in under65 age group colon tissue (a1, a2). Kaplan-Meier survival & cox-regression results (b1, b2) for the right and left side discrimination of the group with poor survival information.
(PDF)

S8 Fig Kaplan-Meier survival & cox-regression results in over65 age group colon tissue (a1, a2). Kaplan-Meier survival & cox-regression results (b1, b2) for the right and left side discrimination of the group with poor survival information.
(PDF)

S1 Table Number of patients with right-left side expression data. (DOCX)

S2 Table Number of patients with expression data by age and gender. (DOCX)
