# Supplementary figures and images for: Could molecular variations be predictive in right and left colon cancer in different gender and age groups?
Source: PLoS One. 2026 Jul 10;21(7):e0351228. doi: 10.1371/journal.pone.0351228 (PMC13353988; doi:10.1371/journal.pone.0351228)

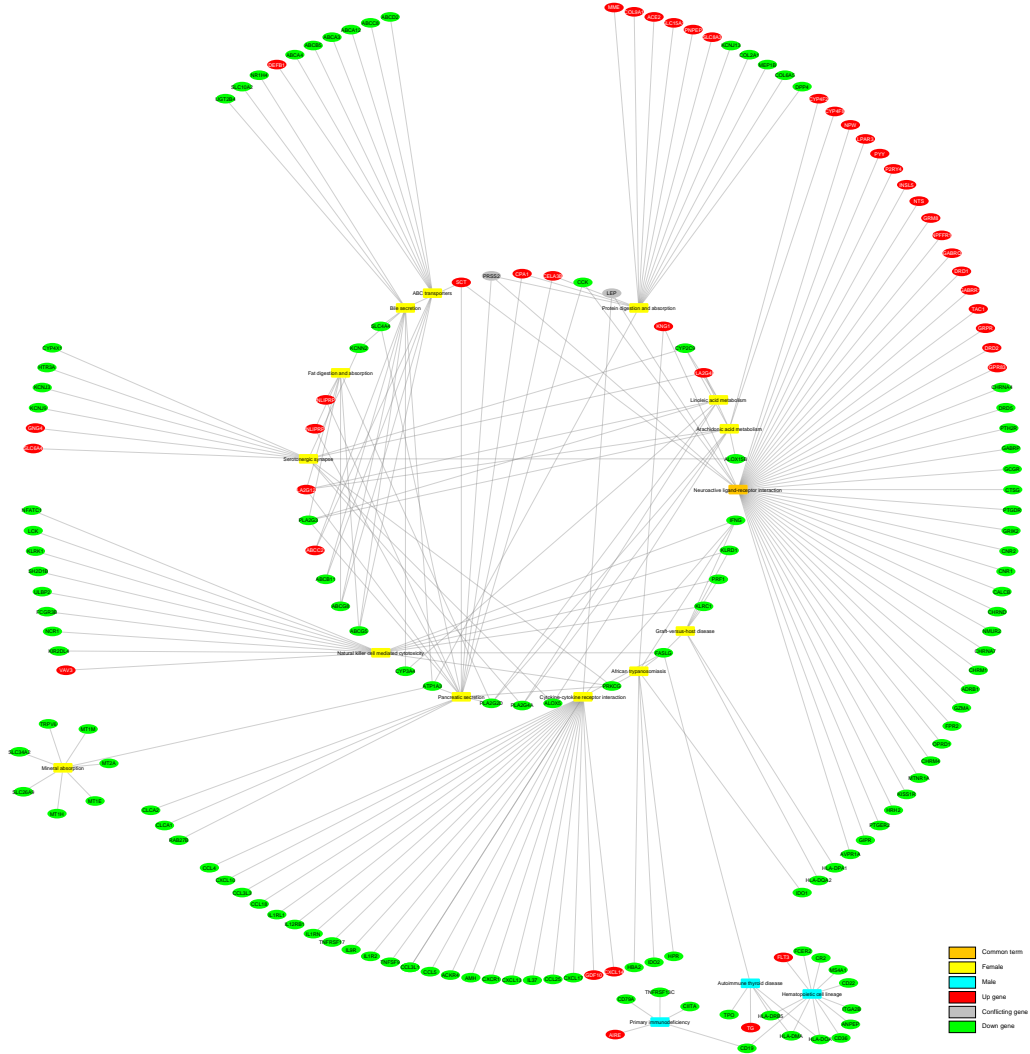

Supplement: S1 Fig — (PDF) [file pone.0351228.s001.pdf]

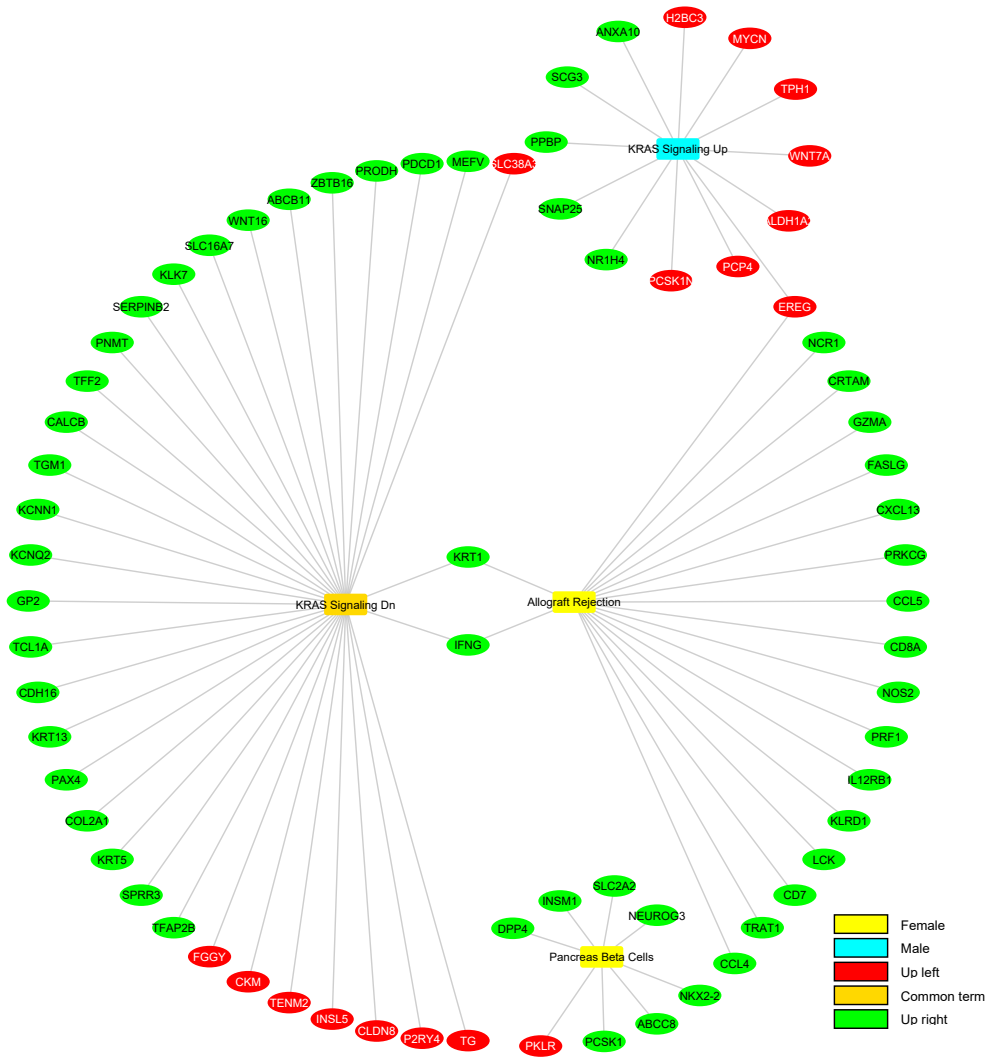

Supplement: S2 Fig — (PDF) [file pone.0351228.s002.pdf]

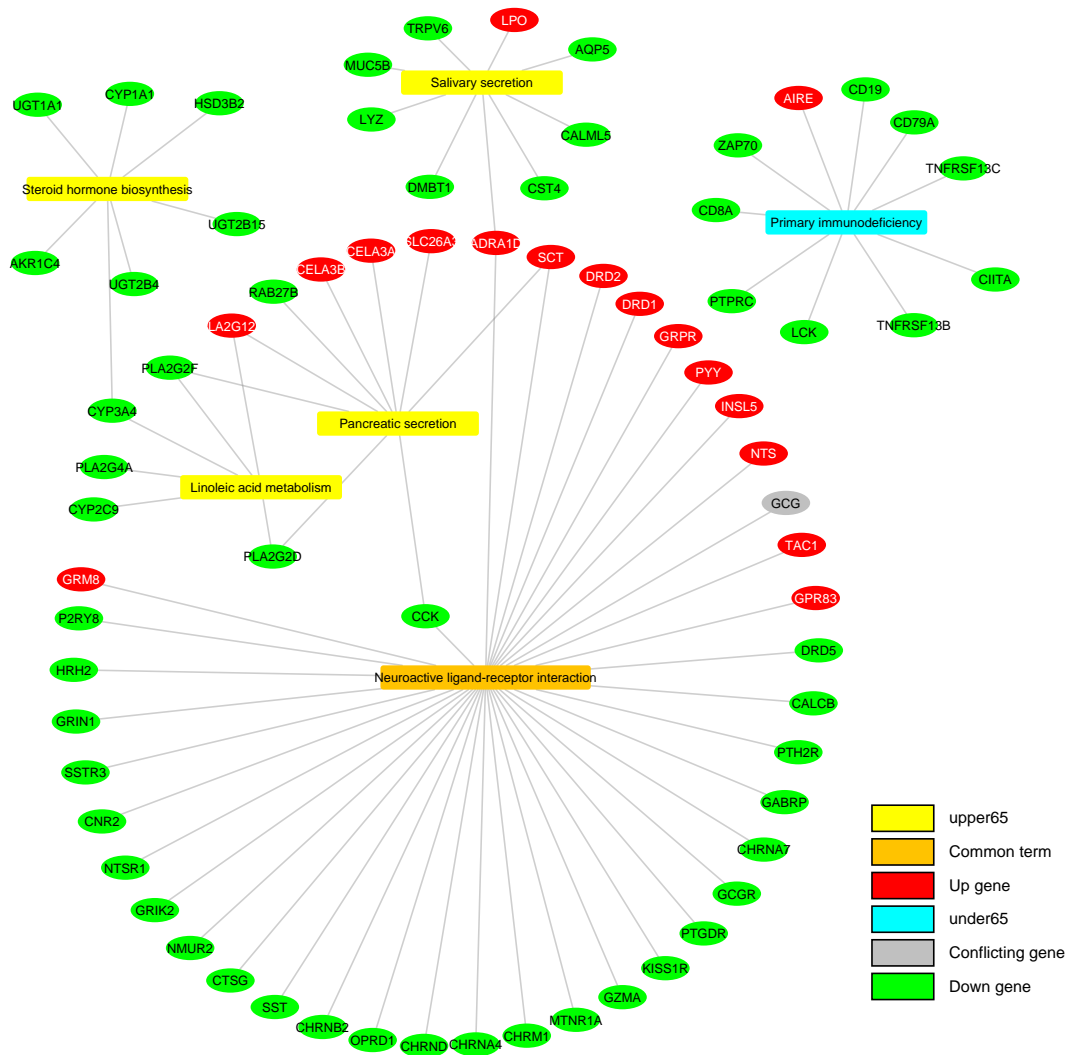

Supplement: S3 Fig — (PDF) [file pone.0351228.s003.pdf]

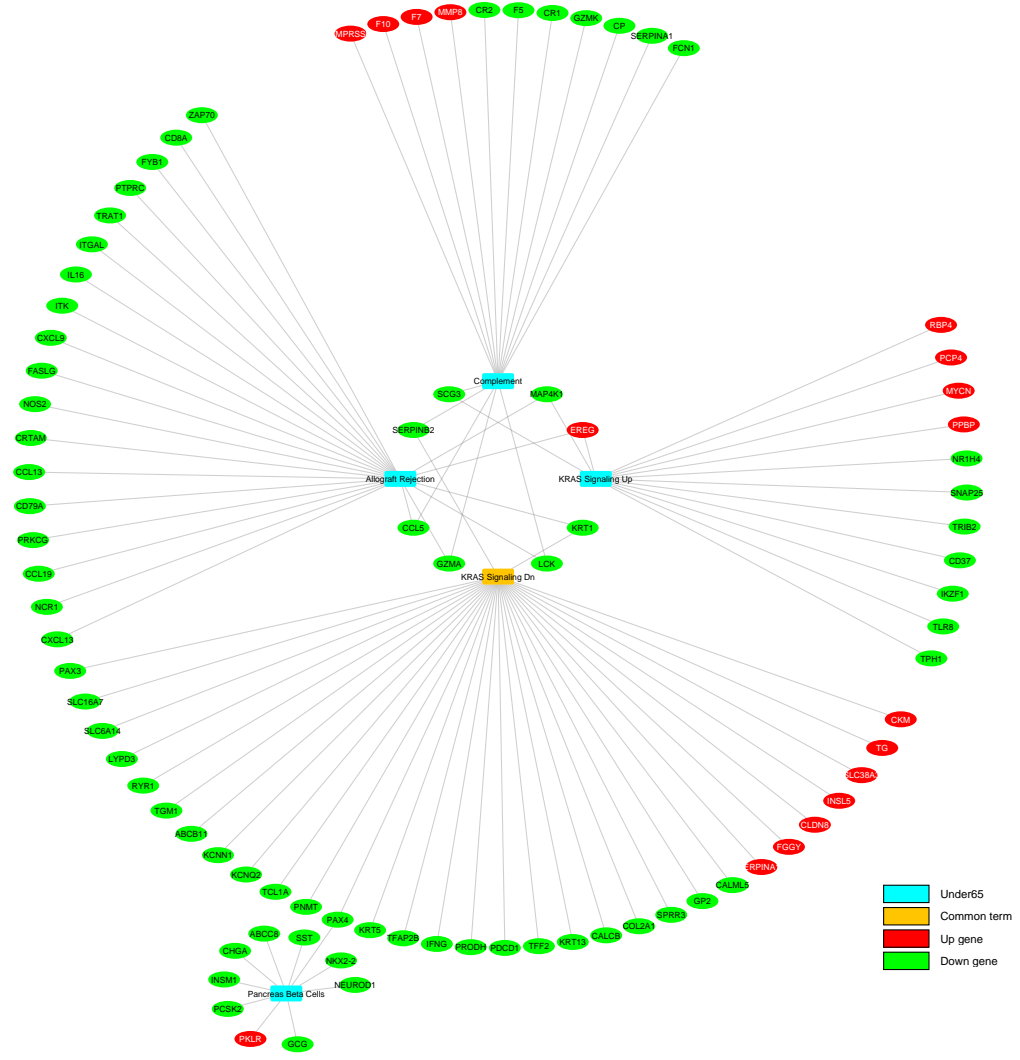

Supplement: S4 Fig — (PDF) [file pone.0351228.s004.pdf]

Survival analysis results for male

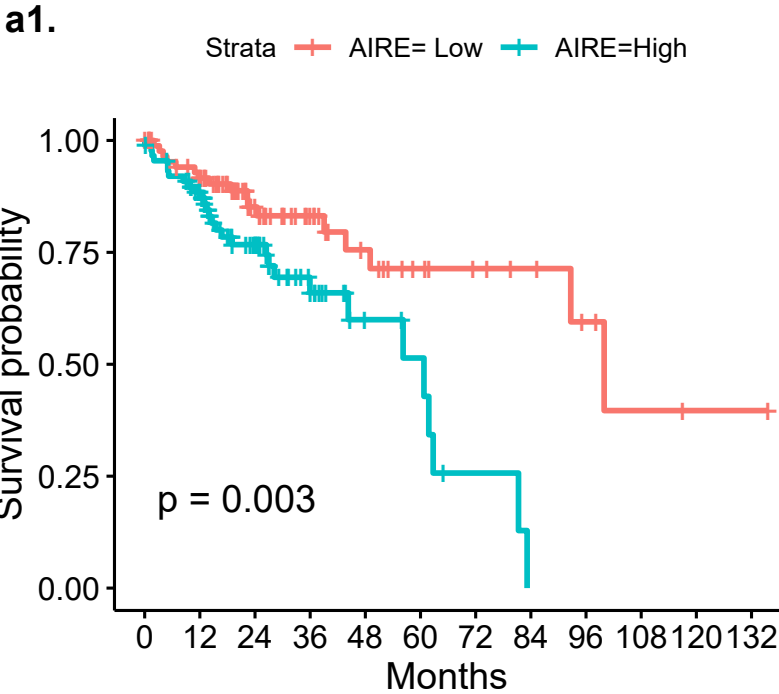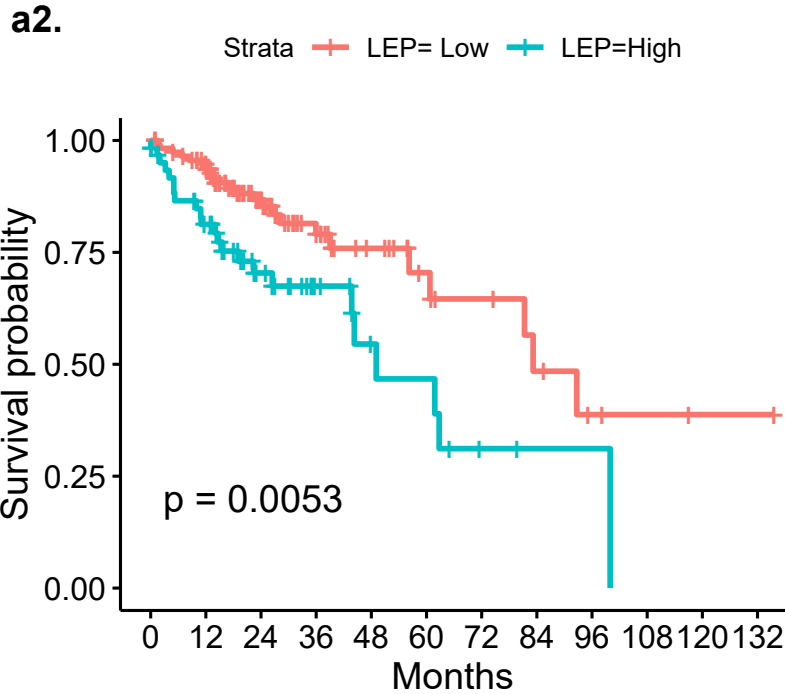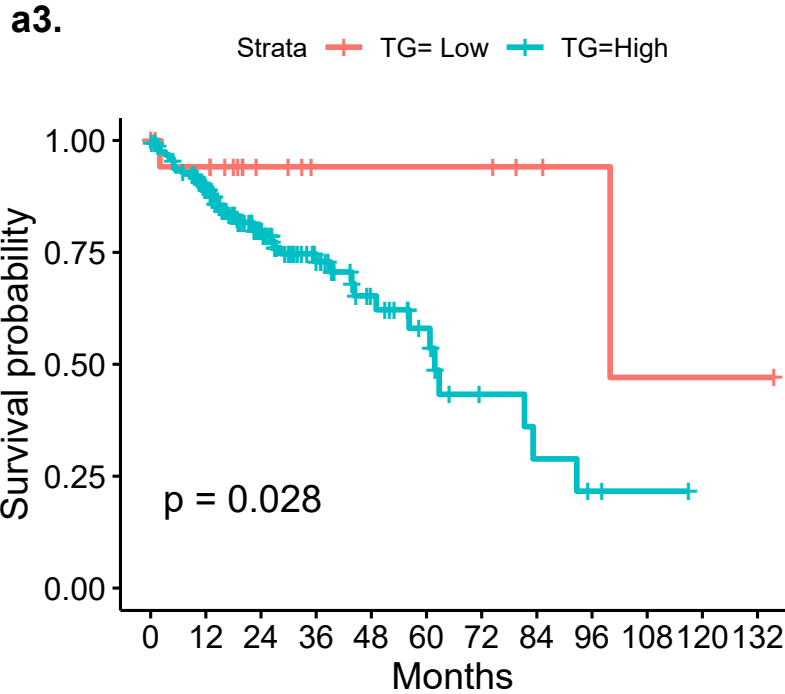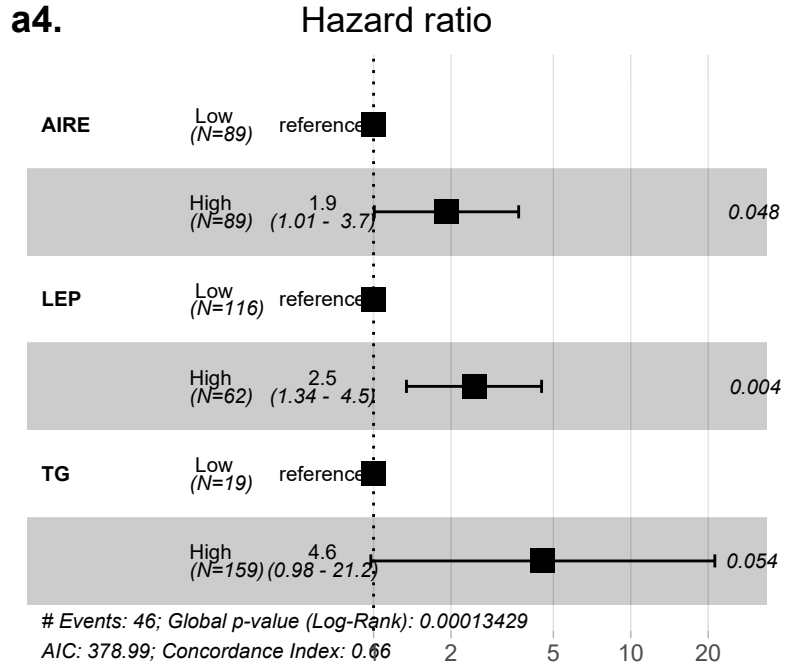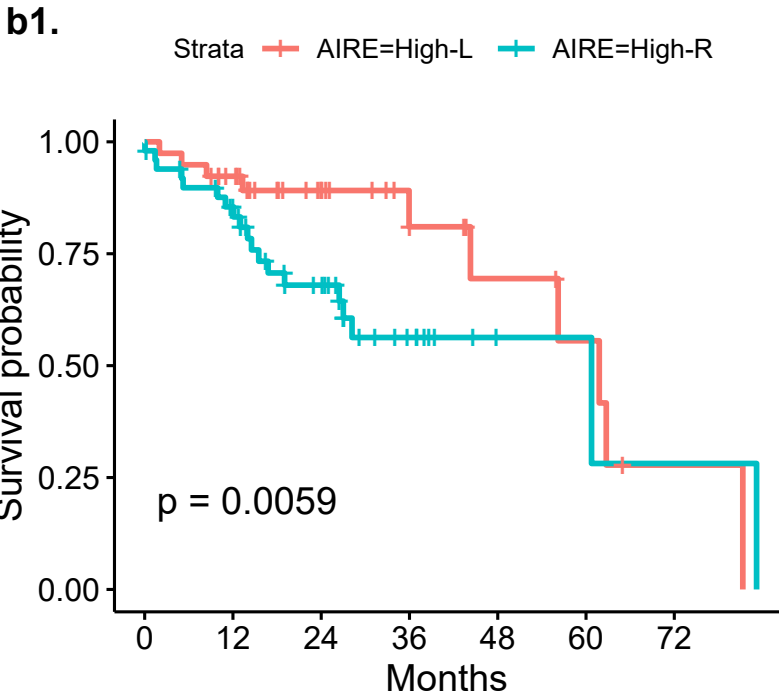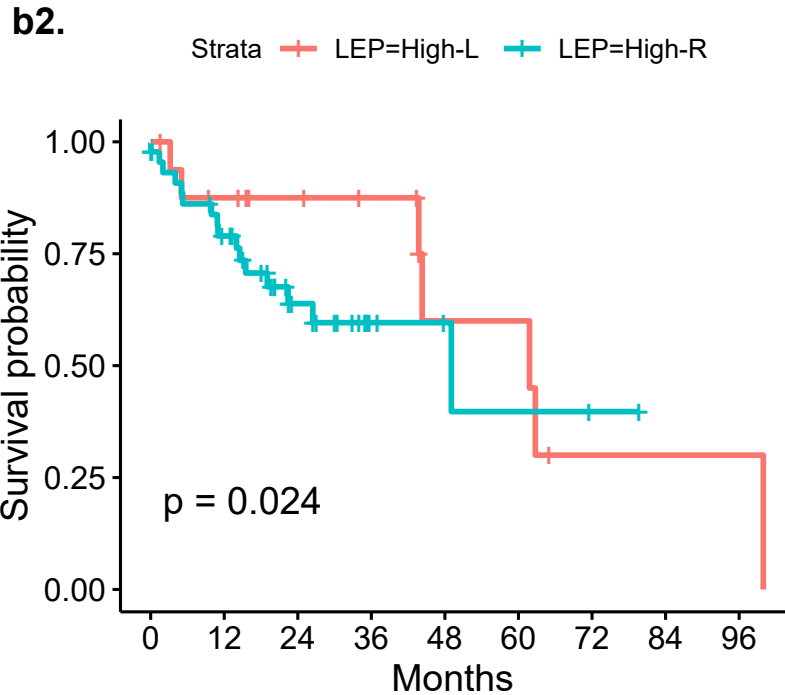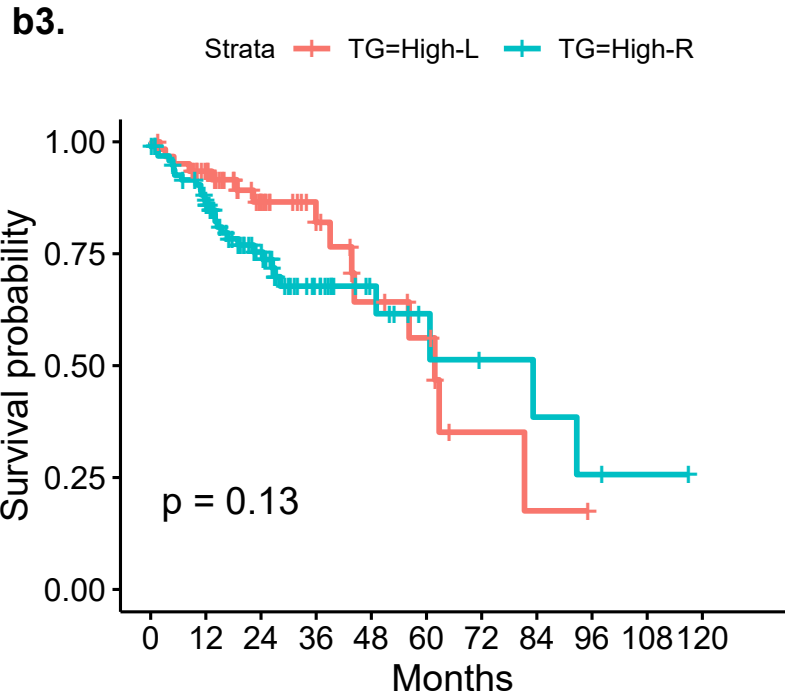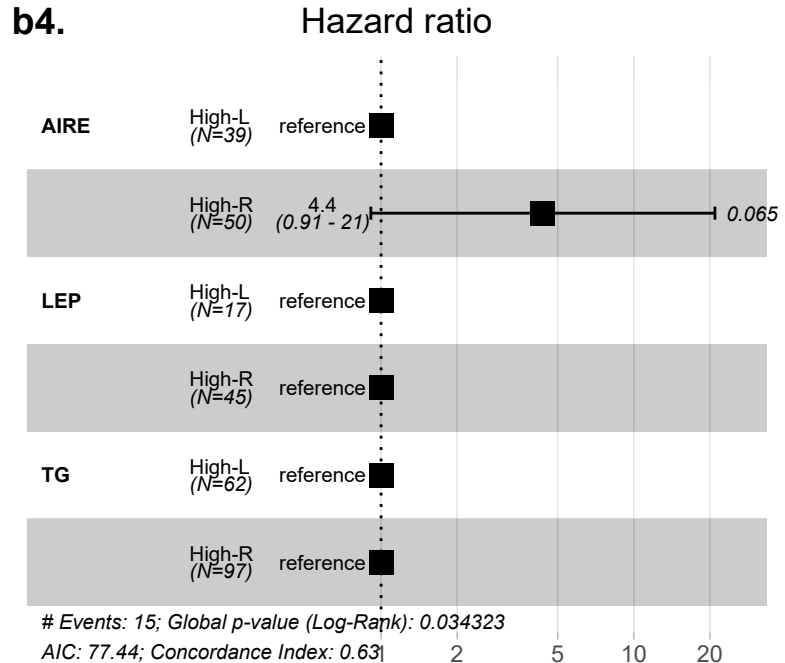

Supplement: S5 Fig — Kaplan-Meier survival & cox-regression results (b1, b2, b3, b4) for the right and left side discrimination of the group with poor survival information. (PDF) [file pone.0351228.s005.pdf]

Survival analysis results for female

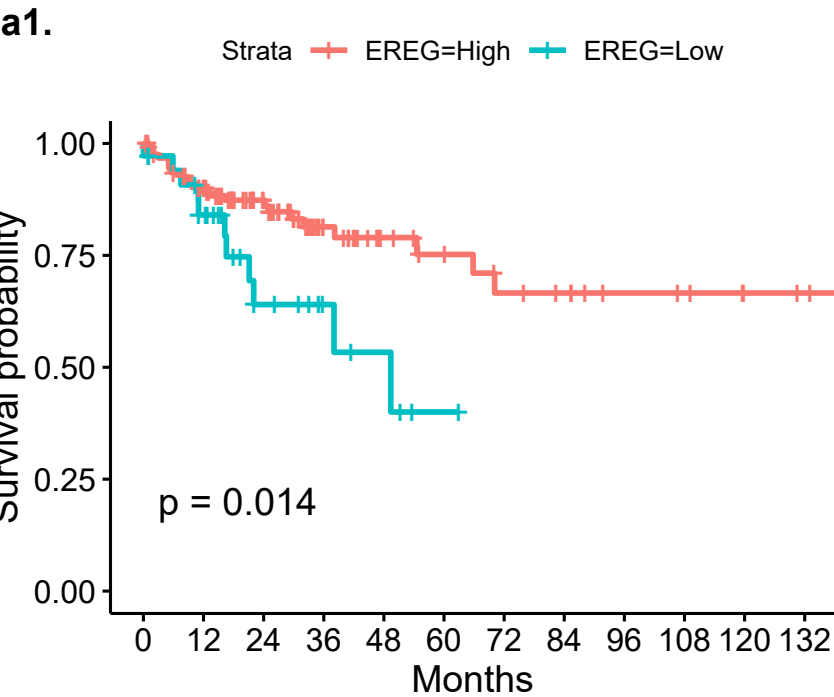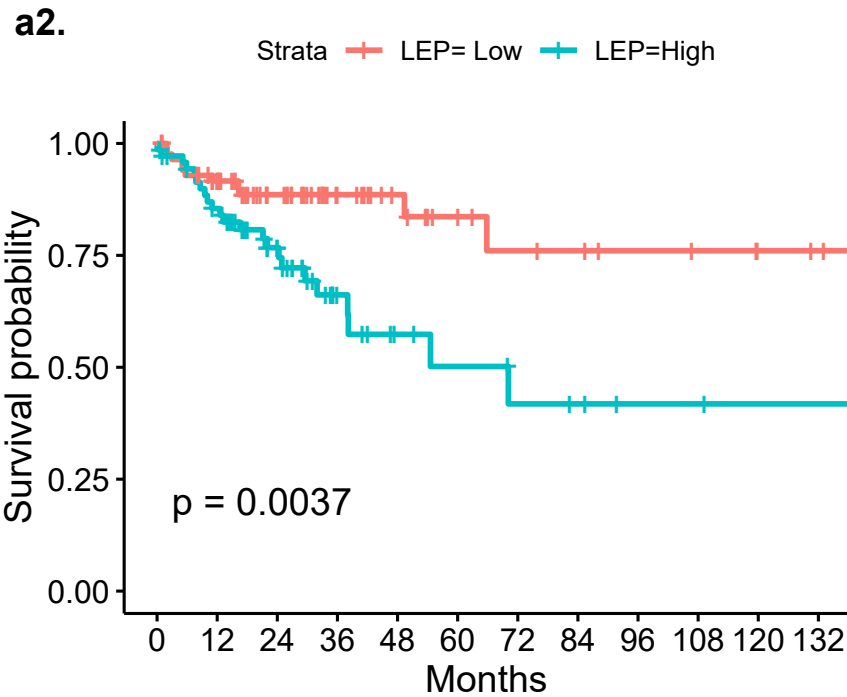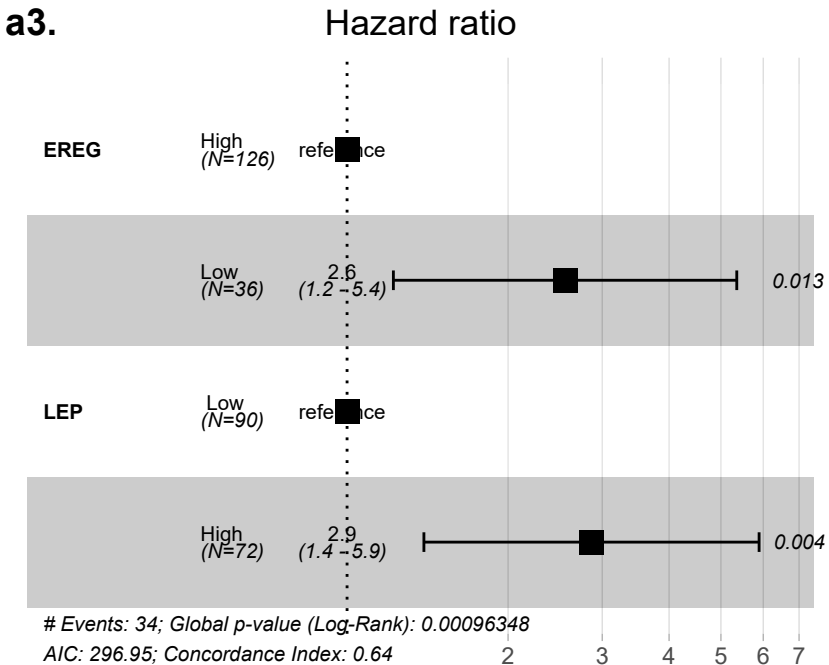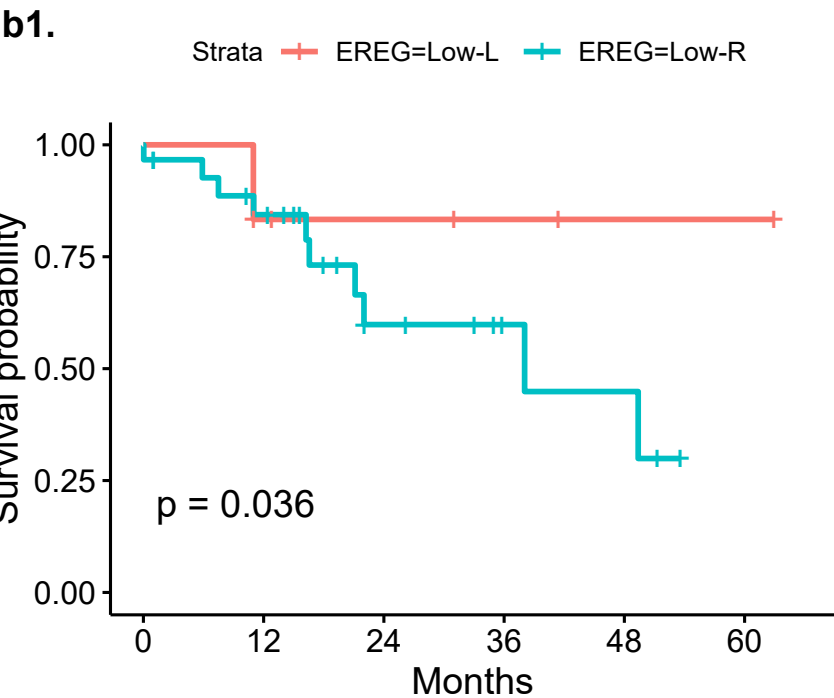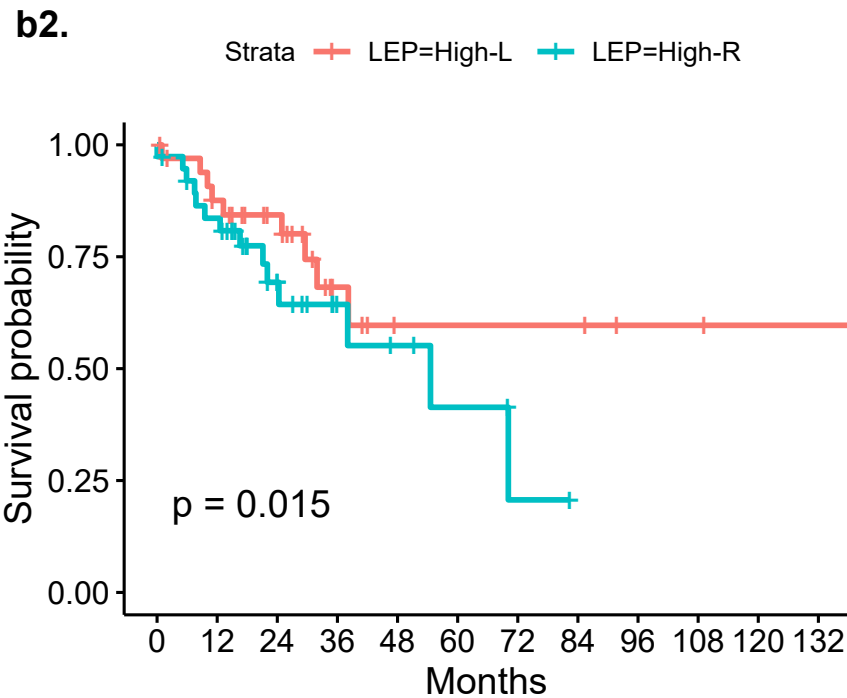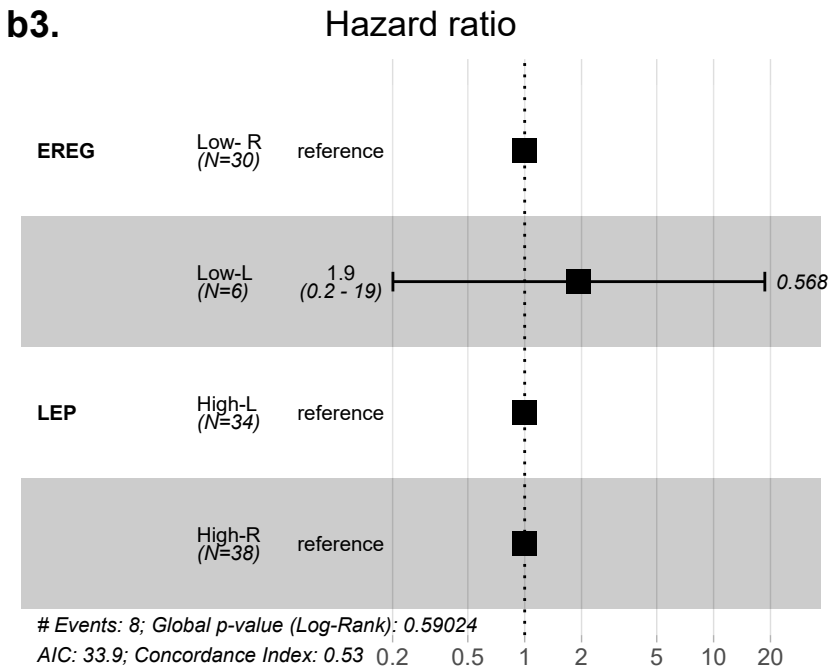

Supplement: S6 Fig — Kaplan-Meier survival & cox-regression results (b1, b2, b3, b4) for the right and left side discrimination of the group with poor survival information. (PDF) [file pone.0351228.s006.pdf]

# Survival analysis results for under 65 age

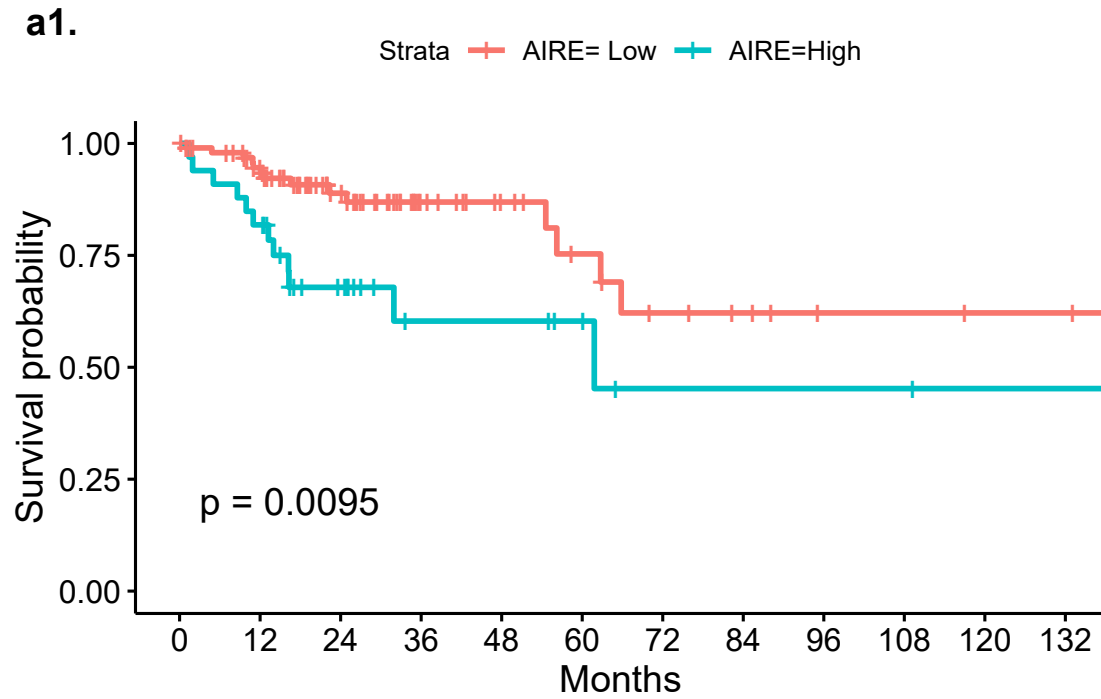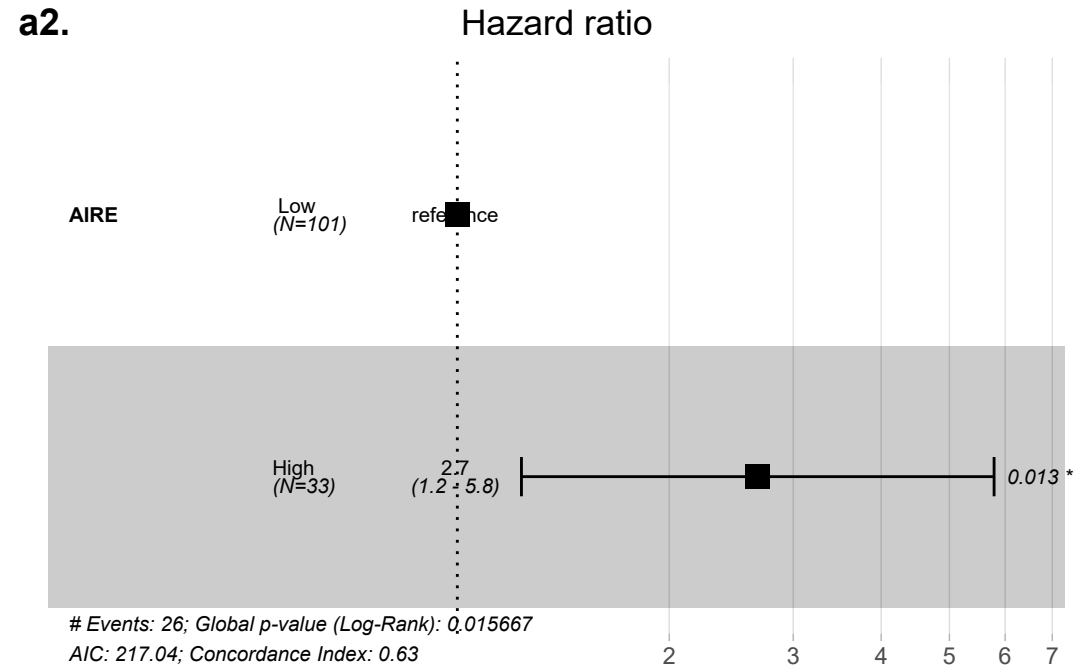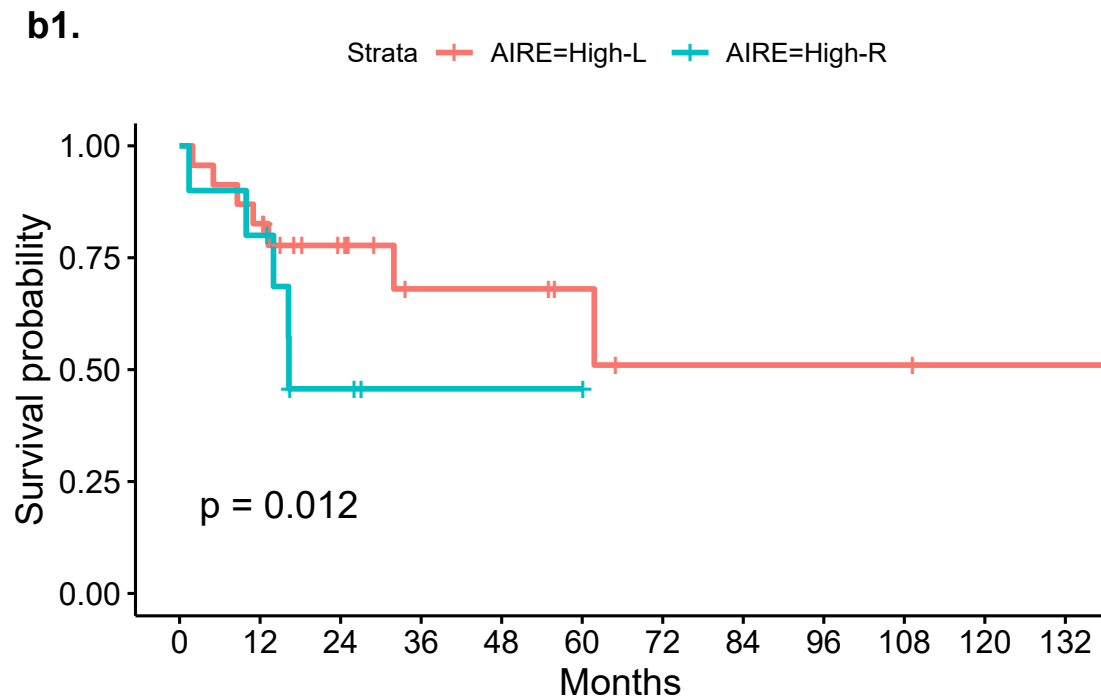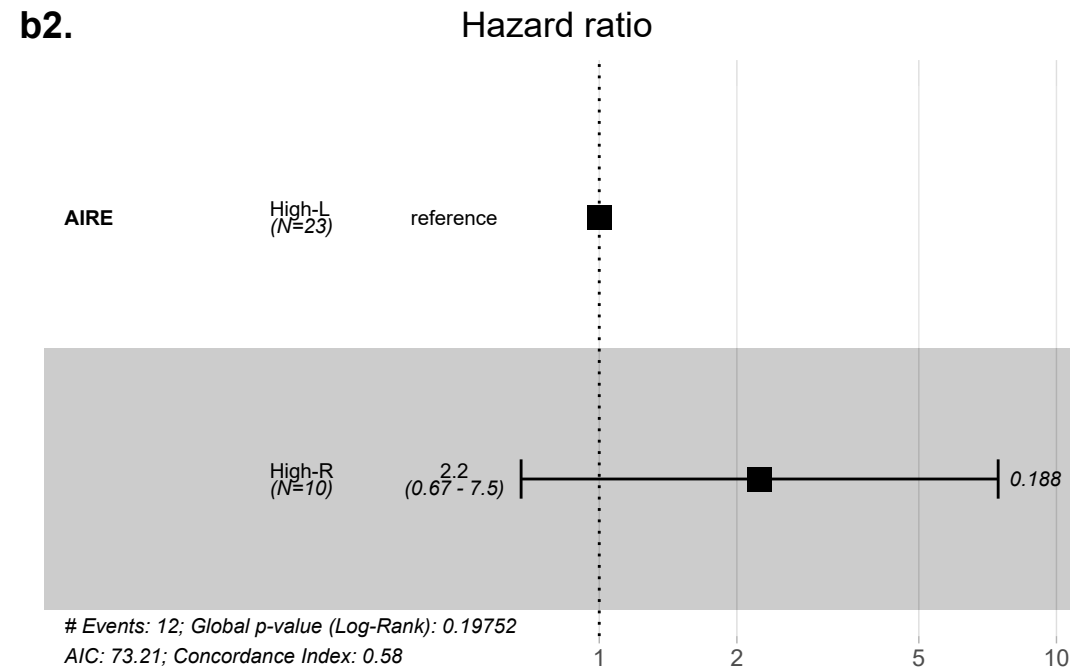

Supplement: S7 Fig — Kaplan-Meier survival & cox-regression results (b1, b2) for the right and left side discrimination of the group with poor survival information. (PDF) [file pone.0351228.s007.pdf]

# Survival analysis results for upper 65 age

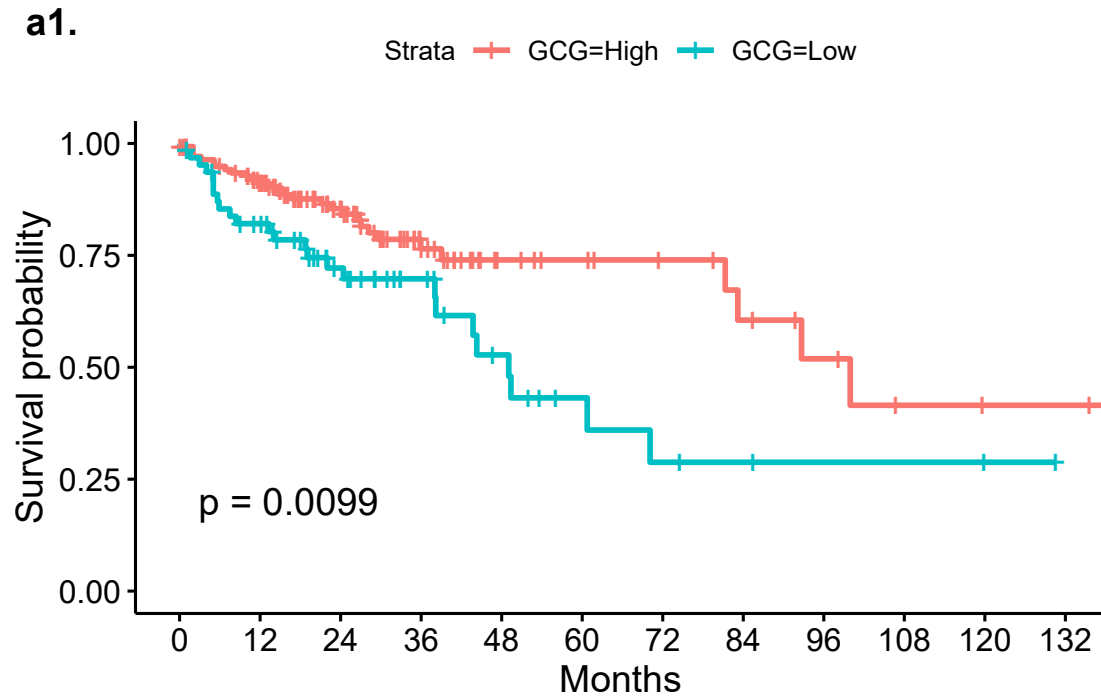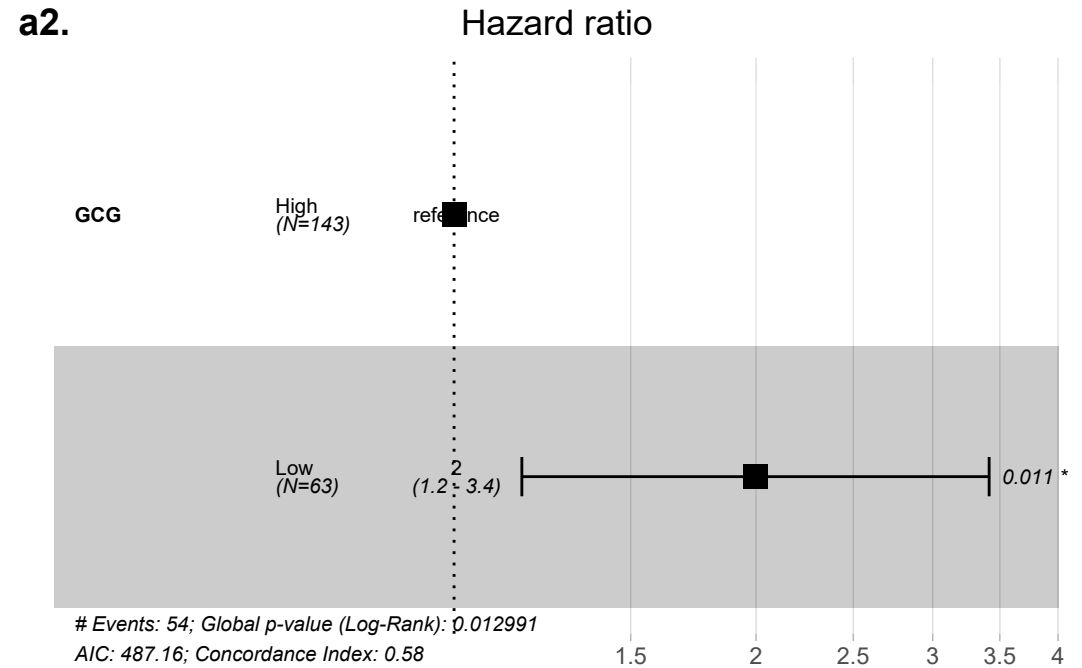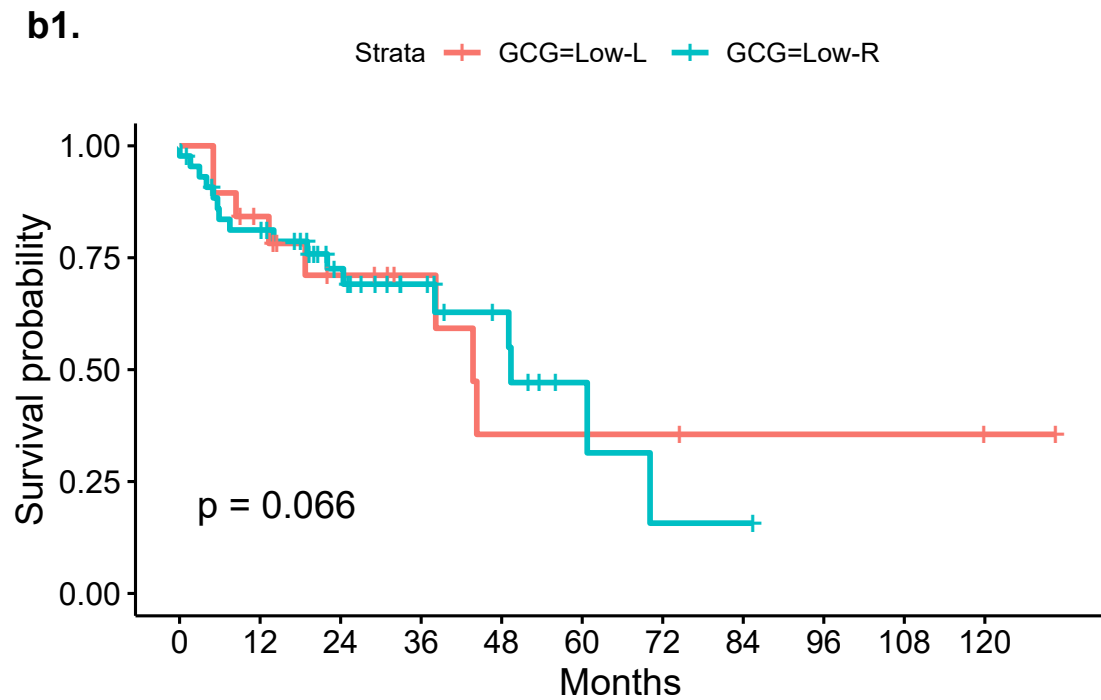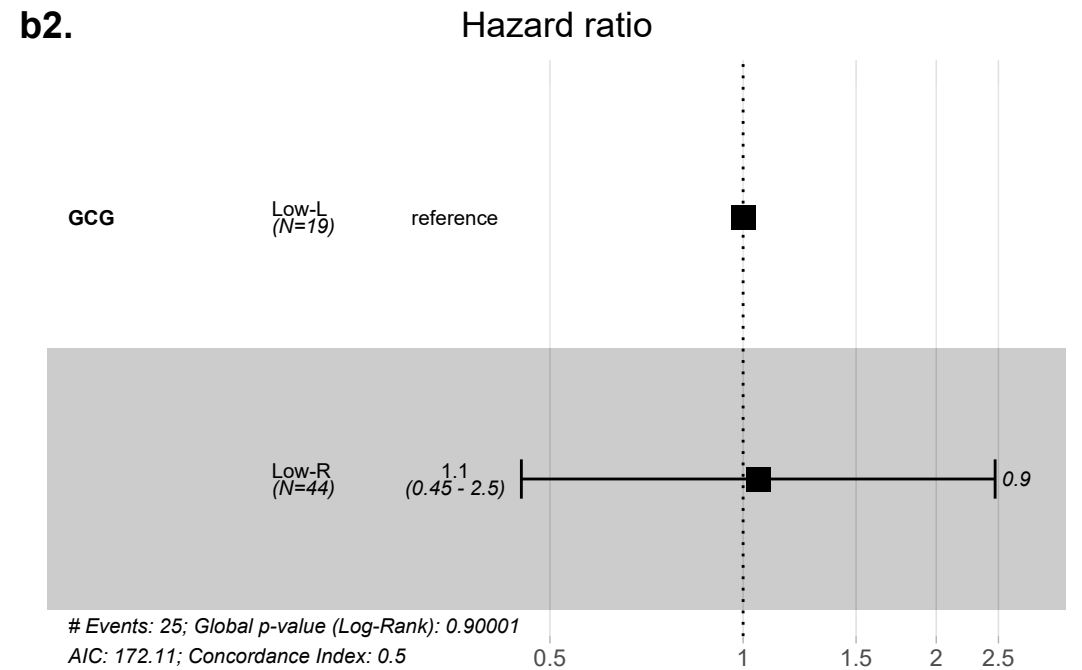

Supplement: S8 Fig — Kaplan-Meier survival & cox-regression results (b1, b2) for the right and left side discrimination of the group with poor survival information. (PDF) [file pone.0351228.s008.pdf]

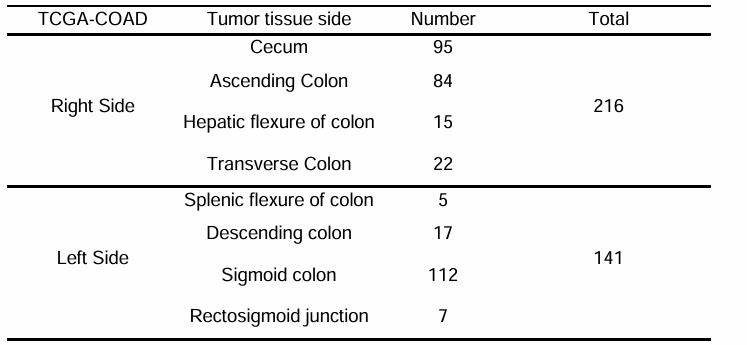

Supplement: S1 Table — (DOCX) [file pone.0351228.s009.docx]

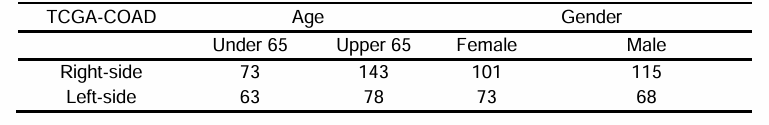

Supplement: S2 Table — (DOCX) [file pone.0351228.s010.docx]
